# Lower Left Thalamic Myo-Inositol Levels Associated with Greater Cognitive Impulsivity in Marijuana-Dependent Young Men: Preliminary Spectroscopic Evidence at 4T

**DOI:** 10.4172/2155-6105.S4-009

**Published:** 2013-03-20

**Authors:** Yasmin Mashhoon, J Eric Jensen, Jennifer T Sneider, Deborah A Yurgelun-Todd, Marisa M Silveri

**Affiliations:** 1 McLean Hospital/Harvard Medical School, 115 Mill St., Belmont, MA 02478, USA; 2 Brain Institute, University of Utah School of Medicine, Salt Lake City, UT 84108, USA

**Keywords:** Impulsivity, Marijuana, Myo-Inositol, Proton, Spectroscopy, Thalamus, Neurochemistry

## Abstract

The effects of chronic marijuana (MRJ) use on neurochemistry are not well characterized. Previously, altered global myo-Inositol (mI) concentrations and distribution in white matter were associated with impulsivity and mood symptoms in young MRJ-dependent men. The objective of this study was to retrospectively examine previously collected data, to investigate the potential regional specificity of metabolite levels in brain regions densely packed with cannabinoid receptors. Spectra were acquired at 4.0 Tesla using 2D *J*-resolved proton magnetic resonance spectroscopic imaging (MRSI) to quantify the entire *J*-coupled spectral surface of metabolites from voxels in regions of interest. For the current regional spectral analyses, a 2D-JMRSI grid was positioned over the central axial slice and shifted in the *x* and *y* dimensions to optimally position voxels over regions containing thalamus, temporal lobe, and parieto-occipital cortex. MRJ users exhibited significantly reduced mI levels in the left thalamus (lThal), relative to non-using participants, which were associated with elevated cognitive impulsivity. Other regional analyses did not reveal any significant group differences. The current findings indicate that reduced mI levels are regionally specific to the lThal in MRJ users. Furthermore, findings suggest that mI and the lThal uniquely contribute to elevated impulsivity.

## Introduction

Cannabis, or marijuana (MRJ), is the most commonly used illicit drug in the United States, with regular MRJ consumption being particularly prevalent among adolescents and young adults [1]. Early initiation of MRJ use can increase the risk of long-term dependence [2] and also disrupt adolescent neuromaturation processes that produce persistent neurobiological alterations in adult neural circuitry [3]. Moreover, converging lines of neuropsychological and neuroimaging evidence indicate strong associations between chronic MRJ use and impaired cognitive functioning [4], including disruptions in behavioral response inhibition [5], impulsivity [6], and decision-making [7].

Sub-optimal cognitive processing associated with chronic MRJ consumption is consistent with alterations in frontal and medial temporal lobe structure and function. Functional neuroimaging studies in MRJ users have shown patterns of abnormal activation in prefrontal, parietal and cerebellar regions during visual attention tasks [8], and alterations in anterior cingulate cortex (ACC) and dorsolateral prefrontal cortex (DLPFC) function during response inhibition and performance monitoring tasks [5]. Furthermore, MRJ-related structural abnormalities in the hippocampus, amygdala and thalamus have been reported [9-11]. The thalamus is part of an attentional and sensory processing network that includes the parietal lobe and frontal lobe, and has been shown to be vulnerable to long-term MRJ use [12,13]. Effects of chronic MRJ use may therefore interfere with response control and executive function via disruption of thalamo-cortical processing [14].

The major psychoactive component of MRJ is Δ9-tetrahydrocannabinol (Δ9-THC), which acts as an agonist at the CB1 cannabinoid receptor [15]. The densely-expressed CB1 receptor is the only known cannabinoid receptor in the central nervous system and is involved in the regulation of cognition, memory, and motor activity [16]. Dense concentrations of cannabinoid receptor binding sites are located in frontal cortex, temporal cortex, hippocampal formation, occipito-temporal gyrus, as well as the parietal and occipital cortices [16,17]. Cannabinoid binding sites also are localized within the thalamus, including the mediodorsal, medioventral, and anterior thalamic nuclei that are connected to forebrain regions involved in cognitive functions [16]. To date, the relationship between protracted THC exposure and neurochemical sequelae in regions like the thalamus and temporal cortex, which are critically involved in mediating executive cognitive function, has not been well characterized.

Proton magnetic resonance spectroscopy (^1^H MRS) is a non-invasive technique for the characterization and measurement of *in vivo* brain metabolites. *N*-acetyl-aspartate (NAA) is one of the most prominent peaks and reflects neuronal integrity, with decreased concentrations indicating neuronal loss and dysfunction [18]. Choline (Cho) reflects signals from multiple choline compounds and is associated with myelination and cellular membrane turnover [19]. The creatine (Cr) signal includes concentrations of intracellular Cr and phosphocreatine (PCr), providing a measure of bioenergetic metabolism [20]. By actively facilitating energy transport and metabolism, Cr plays a key role in maintaining steady brain energy production and consumption levels [20,21]. Myo-Inositol (mI) is an astroglial marker that aids in the regulation of cell volume, neuronal metabolism and energy consumption [22, 23]. Other prominent metabolites, albeit at a lower limit of detection, include excitatory neurotransmitter glutamate (Glu), glutamine (Gln), a glial precursor to Glu, and inhibitory neurotransmitter gamma amino-butyric acid (GABA). Detection and quantification of these peaks are better resolved at high field and benefit from the use of specialized spectroscopic sequences.

To date, the neurochemical effects of long-term MRJ use have not been fully elucidated, with only a limited number of investigations utilizing ^1^H MRS to assess cellular health and integrity in MRJ users. Chang and colleagues found elevated thalamic Cr and lower mI, Cho and Glu in the basal ganglia of HIV-negative chronic MRJ users, suggestive of neuronal and glial dysfunction [24]. Hermann et al. reported diminished NAA levels in the DLPFC of MRJ users, suggestive of reduced neuronal and axonal integrity in this frontal lobe region resulting from chronic MRJ exposure [25]. Adolescent MRJ users exhibit significant reductions in ACC Glu, NAA, Cr, and mI, reflecting altered glutamatergic neurotransmission and neuronal integrity, as well as changes in ACC energetic and glial metabolism [26]. In our study utilizing ^1^H magnetic resonance spectroscopic imaging (MRSI) with tissue segmentation at 4T, alterations in global mI concentrations and mI distribution in white matter (WM), but not GM, were observed, and were associated with changes in impulsivity and mood symptoms in the MRJ-dependent group [27].

While the earlier study evaluated group differences in global levels of proton metabolites in GM and WM across a large matrix of voxels positioned in the medial temporal cortex [27], the primary objective of the present study was to perform a new analysis using this matrix to evaluate regional proton metabolite differences in this previously studied cohort. Spectra were extracted from voxels positioned in the thalamus (Thal), the temporal cortex (TC) and the parieto-occipital cortex (POC), which are regions with known moderate-to-high cannabinoid receptor binding density that could be isolated within the MRS matrix. Group metabolite differences in these regions were assessed as potential neurobiological correlates of impulsivity and mood measures. Examining alterations in molecular mechanisms following long-term MRJ use that may relate to changes in cognitive function is of clinical importance and will also begin to resolve the paucity of research in this area. It was hypothesized that MRJ users would exhibit decreased mI levels in the Thal and TC, relative to the POC and as compared to non-using participants. MRJ users were also hypothesized to demonstrate significant relationships between altered regional levels of mI, mood and impulsivity.

## Materials and Methods

### Participants

Thirteen men diagnosed with MRJ-dependence (MRJ; age=21.3 ± 3.6 years, education=13.5 ± 1.9 years, 2 left-handed) and ten healthy non-using men (NU; age=24.7 ± 4.9 years, education=15.8 ± 2.5 years, 0 left-handed) constituted the study groups. MRJ-using participants represent a subset from the previous study [27], as metabolite spectra of insufficient quality precluded inclusion of some previous participants in the current regional analysis. Clinical assessments were conducted using the Structured Clinical Interview for DSM-IV Non-Patient Edition (SCID-1/NP), which is widely used to reliably determine Axis I disorders in clinical populations. NU participants were recruited from the local community, were free of Axis I psychiatric diagnoses based on SCID assessments, and reported no psychoactive substance use other than minimal alcohol use, averaging 3.9 ± 2.9 alcoholic drinks per week. MRJ users met DSM-IV criteria for MRJ dependence and were active MRJ users, with an average age of first MRJ use=15.9 ± 2.2 years and duration of MRJ use=5.4 ± 2.8 years, and reported currently using MRJ 5.6 ± 1.7 times per week and consuming 16.7 ± 18.8 alcoholic drinks per week. Exclusion criteria for all participants included DSM-IV Axis I diagnoses (other than marijuana dependence in the MRJ group), current psychoactive substance use, history of organic mental disorder, head trauma, loss of consciousness, seizure disorder or central nervous system disease, or contraindications to scanning. All participants completed a clinical MRI scan, which was read and interpreted by a clinical neuroradiologist. No clinical brain abnormalities were present in any of the study participants.

### Procedure

The Institutional Review Board of McLean Hospital approved all aspects of the clinical research protocol. Following complete description of the study, all participants provided written informed consent and received compensation for participating in the study. Participants provided a urine sample, under the direct observation of the study coordinator, which was tested for tetrahydrocannabinol (THC), amphetamines/methamphetamines, barbiturates, benzodiazepines, cocaine, opiates, phencyclidine, propoxyphene and tricyclic antidepressants (Triage^®^ Drugs of Abuse Panel: Immediate Response Diagnostics, San Diego, CA, USA). Drug panel results confirmed recent MRJ use in MRJ users and no drug use in NU participants. An aliquot of the urine sample from MRJ users was sent for a standard laboratory urinalysis, which included gas chromatography-mass spectroscopy to quantify nor-9-carboxy-delta 9-tetrahydrocannabinol levels (Quest Diagnostics, Cambridge, MA, USA). THC was normalized to urinary creatinine (Cre) for each subject, in order to correct for individual differences in urine concentration. Average THC/Cre levels observed in MRJ users were 439.9 ± 478.7 ng/mg.

### Clinical measures

All participants completed the Barratt Impulsiveness Scale (BIS-11, [28]), the Profile of Mood States (POMS; [29]), the Positive Affect Negative Affect Schedule (PANAS; [30]), and the Beck Depression Inventory (BDI-II; [31]).

### Spatial and spectral localization

*In vivo* proton MRS scans were performed on a full-body 4.0 Tesla Varian Unity/Inova MRI/MRS scanner (Varian, Inc., Palo Alto, CA, USA) and a volumetric TEM design (Bioengineering, Minneapolis, MN, USA) RF head coil operating at 170.3 MHz. A rapid two-dimensional (2D) gradient-recalled echo imaging sequence (12 s) acquired sagittal, coronal, and axial images for rapid determination of head position within the coil. The global magnetic field was manually shimmed using first and second-order shim coils (x, y, z, z2, xy, yz, xz, and x2y2), resulting in unfiltered water line widths ≤ 25 Hz. High-contrast, T1-weighted sagittal and axial images were then acquired (repetition time/echo time (TR/TE)=6.2/11.4 ms, field of view (FOV) (in-plane)=24 × 24 cm, slice thickness=2.5 mm (sagittal 16 slices, axial 32 slices), matrix size=256 × 256) to guide placement of a 4 cm thick point-resolved spectroscopy (PRESS) box inferior to the body of the corpus callosum over the mid-sagittal image, and readjusted in the axial plane to align the top of the box with the top of the caudate. This placement permitted sampling voxels from regions such as the thalamus and the temporal and parietal lobes. Anatomical landmarks identified on the basis of gyral boundaries and structural landmarks visible in the images allowed consistent between-subject voxel placement.

### 2D-JMRSI acquisition

2D *J*-resolved magnetic resonance spectroscopic imaging (*J*-MRSI) combined with standard PRESS selective excitation incrementally acquired phase-encoded spectral data, with increasing TE to sample the *J*-coupling of proton metabolites. Sixteen individual TE-stepped spectra (30 to 330 milliseconds (ms), 20 ms increments) were collected for each of the 96 circular, sparsely sampled k-space points. Spectral acquisition parameters were: TR=1.25 s, sampling matrix=14 × 14 (circular-sparse), spectral bandwidth=2 kHz, complex time-points=1024, FOV=18 × 18 cm, NEX=1, nominal voxel volume=6.6 cc (10.2 cc effective voxel size), total spectral acquisition time=32 min. Fourier-transformation of TE-stepped data in the TE dimension reveals the primary advantage of this approach: *J*-resolved datasets for each voxel. The *J*-resolved technique allows for quantification of the entire *J*-coupled spectral surface, thereby improving the measurement precision of strongly-coupled resonance species, e.g., mI, Glu, and Gln, as compared to single-echo techniques [32].

### Regional 2D-JMRSI voxel positioning and spectral processing

The 96 sparsely sampled k-space points per echo-time were first read into a zero-padded 16 × 16 matrix for all 16 echo-times. The TE-series (16 k-space *J*-MRSI data sets) were zero-filled out to 64 k-space and digitally-apodized prior to Fourier transforming in the TE dimension. Each *J*-resolved, 2D MRSI k-space set was then digitally filtered in kx and ky with a Hanning k-space filter to produce *J*- and spatially-resolved spectra. For each subject, the 2D *J*-MRSI grid was shifted in the x and y dimensions to position a 16 × 16 square matrix of voxels over the volume of interest. Automated software developed on-site was used to omit voxels from the entire *J*-resolved MRSI dataset that contained excessive lipid signal from sub-cranial fat layer.

**Figure 1:**
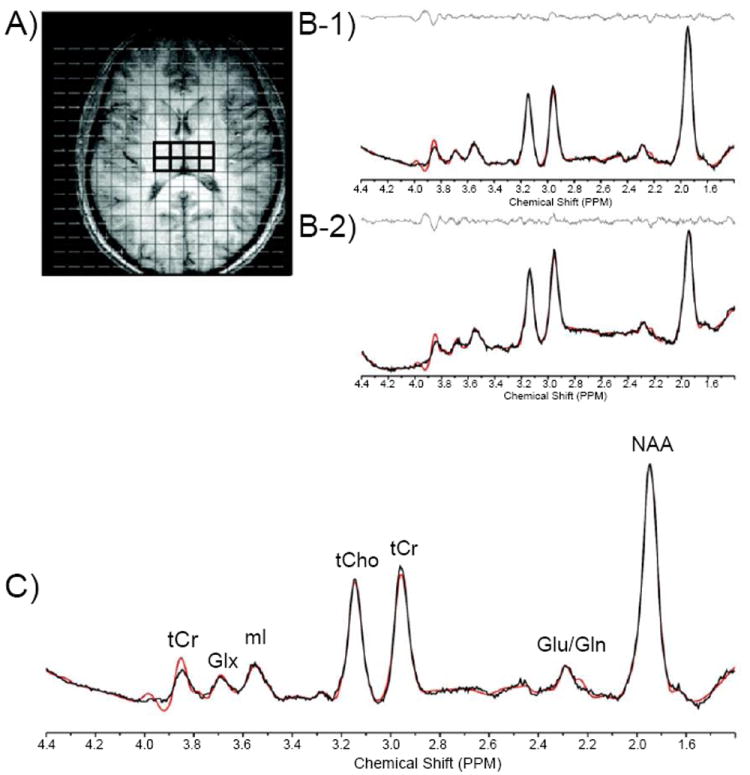
(A-C) A. Axial T1-weighted representative image depicting MRSI grid with voxels positioned in the left and right thalamus, and corresponding J=0.0 Hz sample spectra from voxels in the left (B-1) and right (B-2) thalamus. C. J=0.0 Hz spectrum shown with LCModel fit and labeled metabolites. **Abbreviations:** Cr: Creatine; Cho: Choline; ML: Myo-Inositol; NAA: *N*-acetyl-aspartate; Glu: Glutamate; Gln: Glutamine

**Figure 2:**
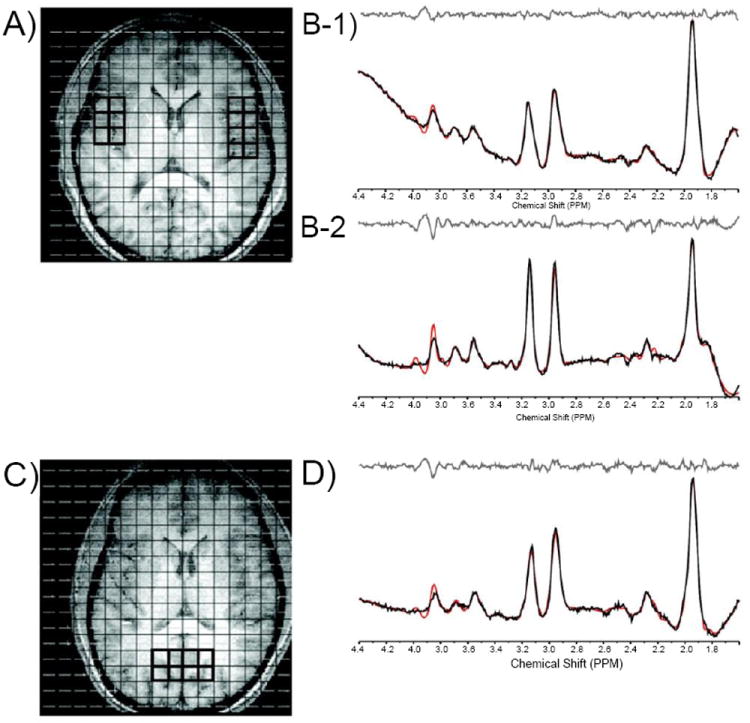
Axial T1-weighted images depicting MRSI grid with voxels positioned in the left and right temporal cortex (A) and in the parieto-occipital cortex (POC; C). Also shown are corresponding *J*=0.0 Hz sample spectra from voxels in the left (B-1) and right (B-2) temporal cortex and in the POC (D).

In order to perform regional analyses, the 2D-*J*MRSI grid was positioned over the central axial slice (slice number 16 or 17 of 32 total) and shifted in the *x* and *y* dimensions in the axial plane to optimally place a matrix of voxels over regions of interest. Voxels positioned over the left (lThal) and right thalamus (rThal) were placed on each side of the midline separated by the putamen (Figure 1). Voxels positioned over the left (lTC) and right temporal cortex (rTC) were placed over GM (Figures 2A, 2B-1 and 2B-2). Voxels were also placed in the POC along the midline (Figures 2C and 2D). Potential partial overlap with some adjacent structures was unavoidable in these regions due to voxel volume and shape. All remaining spectra were then automatically fitted using an optimized, two-dimensional LCModel algorithm produced from GAMMA-simulated basis sets [32], which quantifies all 32 *J*-resolved spectral extractions within a *J*-bandwidth of 25 Hz and provides two-dimensional integrals for Cho, mI, Glu, Gln, NAA, NAA + N-acetyl-aspartyl-glutamate (NAAG), and Cr. To ensure the quality of spectra that were included in the regional analyses, the *J*=0.0 Hz spectrum and LCModel fit for each voxel was evaluated by an experienced spectroscopist (JEJ). Sample spectra (*J*=0.0 Hz) from lThal voxels, as well as a sample spectrum (*J*=0.0 Hz) with LCModel fit and labeled metabolites are presented in figures 1A-1C). The number of voxels that produced spectra of sufficient quality, which were defined by well-resolved metabolite signals, did not differ significantly between MRJ and NU groups in any region (Table 1). Spectral data from the 2 left-handed MRJ users and 1 NU participant were excluded due to low signal-to-noise and/or poor spectral quality, likely resulting from subject movement.

### Proton metabolite quantification

Metabolite ratios were calculated as raw metabolite integrals relative to total Cr. Cr is commonly used as a denominator in proton spectroscopy studies, given that Cr levels generally reflect less inter-individual variation [33]. To ensure that Cr did not differ between groups and was a reliable and unbiased denominator in the present study, the ratio of Cr to the total integrated proton signal (which did not differ by group in any of the seven regions; Table 1) was derived using all fitted metabolites. Metabolites included Cho, Glu, Gln, mI, NAA, and NAA+NAAG. Some metabolite data in each region were excluded due to poor spectral quality, resulting from overlap with adjacent structures and interference from subcutaneous fat and lipids of the skull that may have reduced signal intensity. Ratios of Cr/total signal did not differ between groups in any region except in the rTC (p=0.05; Table 1). In the absence of systematic changes in Cr in multiple brain regions across study groups, all proton metabolites reported were expressed as raw integral values divided by Cr.

### Tissue segmentation

T_1_-weighted image sets were segmented using the automated commercial software package FSL 4.1 (FIMRIB Software Library, Analysis Group, FIMRIB, Oxford, UK) into four individual tissue compartments. The four compartments included GM, WM, cerebrospinal fluid (CSF), and subcortical deep GM (SDGM). To provide partial tissue estimates for each voxel, binary-segmented tissue image sets were convolved with the calculated two-dimensional point-spread function, including digital *k*-space filtering effects. Per voxel, percent relative cortical GM tissue content was estimated by adding raw GM and SDGM and dividing the total GM fraction by the total tissue sum [(GM fraction + SDGM fraction)/(GM fraction + SDGM fraction + raw WM fraction)] × 100%.

### Statistical analyses

Proton metabolites and GM and WM tissue segmentation in the left and right thalamus and left and right temporal cortex were analyzed using repeated measures analyses of covariance (ANCOVAs) to compare groups and test for laterality. Proton metabolites and GM and WM tissue segmentation in the midline POC region were analyzed using one-way ANCOVAs to compare MRJ and NU groups. Clinical measures were analyzed using one-way ANCOVAs to compare MRJ and NU groups. All ANCOVAs included age and education as covariates. Correlations between clinical variables and metabolites were examined using Pearson’s r correlation coefficients. Data were analyzed using SPSS 19.0 (SPSS, Chicago, IL, USA) with a statistical significance threshold of p<0.05.

## Results

### Clinical measures

The results of the clinical measures in this subset of subjects are consistent with those reported for the entire previously studied cohort [27]. MRJ users reported significantly higher cognitive (F[1,21]=7.72, p=0.01), motor (F[1,21]=5.30, p=0.03), and overall impulsivity (F[1,21]=9.06, p=0.01) scores on the BIS-11, relative to NU participants. There was no significant group differences on mood measured using the POMS, PANAS, or BDI-II.

### Tissue segmentation

There were no significant group differences between the relative percentages of GM and WM in any of the MRSI voxels examined (Table 2).

### Proton metabolite ratios

**Figure 3:**
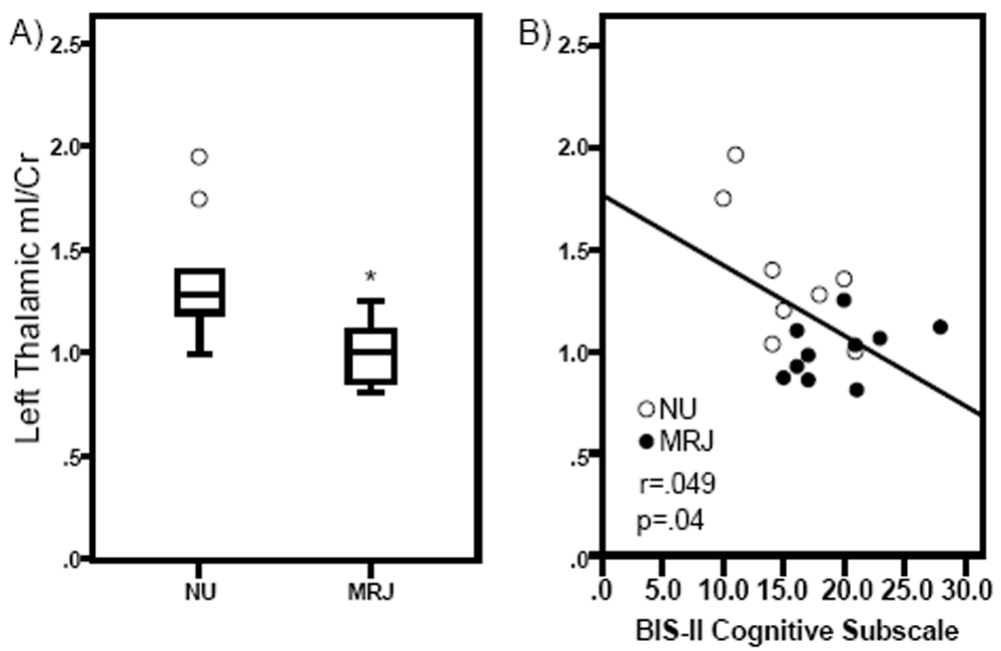
Myo-Inositol (mI)/Creatine (Cr) ratios in the left thalamus (lThal) in NU and MRJ-using groups are shown. A. Significant group differences in mI/Cr ratios. Values are the means ± SD. *p=0.05 relative to NU lThal mI/Cr ratios. B. Scatterplot showing significant correlation between mI/Cr ratios and reported BIS-11 cognitive impulsivity subscale scores.

Analyses of thalamic metabolite levels revealed a significant group × hemisphere interaction (*F*[1,15]=4.53, p=0.05; ES=0.78; Figure 3A), indicating that left Thal mI/Cr ratios were significantly lower in MRJ users relative to NU comparisons. Right Thal mI/Cr levels and other regional analyses of metabolite ratios did not reveal any significant differences between groups (Table 1).

### mI, clinical measures, and marijuana use

Lower left Thal mI/Cr levels predicted greater cognitive impulsivity in MRJ users (*r*=-0.49, p=0.038; Figure 3B), but were not related to the motor and non-planning subscales or overall impulsivity, measured using the BIS-11. However, when mI/Cr levels and cognitive impulsivity ratings were examined separately in MRJ or NU cohorts, no significant relationships were observed. No significant correlations were observed between lThal mI/Cr ratios and any of the mood scale measures (POMS, PANAS, BDI-II). Furthermore, lThal mI/Cr was not associated with current alcohol use, frequency of MRJ use, age of onset or duration of MRJ use or urinary THC/Cr levels.

## Discussion

Findings of the current investigation are consistent with previous reports of lower mI/Cr in the basal ganglia [24] and ACC [26] of young MRJ-users compared to NU participants, substantiating previous global findings [27] to now include lower mI/Cr levels in the lThal. No other significant group differences in metabolite levels were observed in any of the other regions examined. Lower levels of lThal mI/Cr in MRJ-dependent participants were associated with greater cognitive impulsivity on the BIS-11, but no other measures. The cognitive impulsivity subscale of the BIS-11 includes items such as “I like to think about complex problems” and “I am a careful thinker” [28]. A number of studies have utilized the cognitive impulsivity subscale as a reliable construct of rapid decision-making and ability to focus attention (e.g. [28,34]). Overall, the present lThal mI/Cr findings are consistent with the interpretation that mI/Cr levels and cognitive focus are potentially altered by chronic MRJ use [27].

Anandamide (arachidonylethanolamide), an endogenous functional agonist at the endocannabinoid CB1 receptor, is widely distributed in the thalamus [35], suggesting that CB1 receptor-mediated changes in synaptic activity within the thalamus may have direct consequences for the complex transmission of information to the cortex [36]. As THC also acts as an agonist at CB1 receptors, it is possible that chronic MRJ consumption may alter the proper funneling of information through thalamo-cortical networks. In the present study, MRJ-related metabolite differences were regionally specific to the lThal. Asymmetry in thalamic biochemical [37] and functional processing [38] has been previously established. Chronic MRJ use has been shown to interfere with verbal working memory [39], which is a cognitive process hypothesized to be lateralized to left hemispheric regions that includes the left thalamus [40]. Moreover, the left thalamus is also hypothesized to be a critical component of left-brain lateralized cognitive inhibition circuitry [41]. The thalamo-cortical circuit that includes the thalamus and frontal regions such as the DLPFC and ACC is involved in inhibitory processing [14] and is thought to suppress or diminish inappropriate behavioral responses. Left hemisphere dysfunction in regions like the thalamus can interfere with cognitive inhibition circuitry, thereby potentially elevating attention and behavioral problems, such as impulsivity [41]. Thus, future studies should investigate the possibility that left-hemisphere-mediated cognitive functions, such as verbal working memory and behavioral inhibition, and associated left-hemisphere regional metabolite levels are uniquely vulnerable to MRJ-induced dysfunction in male and female cohorts.

Present findings are consistent with previous reports documenting significant relationships between greater levels of impulsivity and MRJ use [6,27,42]. The observed relationships also extend previous findings to suggest that MRJ-related reductions are specific to lThal mI/Cr levels, which were significantly associated with greater cognitive impulsivity. mI is a glial marker that is actively transported into astroglia that are involved in neuronal metabolism, neurotransmitter synthesis, and regulating cellular volume and energy consumption [23]. Receptors for neurotransmitters such as glutamate and dopamine are present on the surface of astroglia, and are therefore directly involved in neural signaling [43,44] and neurotransmission [45]. Reductions in mI levels are suggestive of regionally altered glial metabolism, glial dysfunction, or glial loss [22] in the lThal in MRJ users, and could also reflect altered regional neurotransmission, potentially affecting thalamo-cortical signaling. Disrupted thalamo-cortical signaling may interfere with left-brain lateralized cognitive inhibitory processes by reducing astroglia-mediated energy metabolism [46]. One outcome of diminished inhibitory processing in MRJ users may be elevated cognitive impulsivity. Decreased lThal mI levels in MRJ-dependent participants may also reflect decreased intracellular mI concentration, which could interfere with glial function in neurotrophic signaling and neuroplasticity in that region [47]. The possibility, however, those differences in lThal mI levels and the relationship between lThal mI and cognitive impulsivity may have preceded the onset of MRJ use and/or served catalytically to initiate and maintain MRJ use cannot be ruled out.

There are strengths and limitations of the study that warrant attention when evaluating these preliminary findings. Notable strengths of the study included collection of multiple high field (4T) spectra using 2D *J*-resolved MRSI that were combined with the use of GAMMA-simulated basis sets for LCModel fitting to generate a precise, theoretically-correct template for each spectral extraction. Moreover, the MRSI technique permitted voxels to be accurately positioned in the Thal, TC, and POC and minimized the number of spectra collected with poor signal to noise quality due to interference by subcutaneous lipids. Partial volume effects were also determined from the voxels of interest using tissue segmentation. Lastly, to eliminate any potential limitations associated with self-reported drug use, same day urine samples were collected and tested to determine urinary THC/Cr levels, which serve as a significant objective measure to confirm recent MRJ use.

Given the modest sample size in this study and the inclusion of only male MRJ users, these findings require replication with a larger sample size that includes female MRJ users. Additionally, given that this study is a retrospective analysis of data from an earlier preliminary investigation [27], significance levels for ANCOVAs and correlations were not corrected for multiple comparisons. Notably, a large effect size was achieved for the lThal difference. In addition, post hoc power analyses revealed that only an additional five subjects, yielding a total sample size of 25 subjects, would be needed to survive a conservative Bonferroni correction. MRJ users in the present study were well characterized as young chronic users and clinically diagnosed with marijuana dependence without meeting criteria for any other substance abuse disorders; however, additional studies that include female MRJ users will help to clarify the generalizability of the relationship between lThal mI levels and cognitive impulsivity. Furthermore, future studies should also explore if concurrent alcohol and marijuana dependence produce measurable changes in metabolite levels relative to marijuana dependence alone. In the present study, while the MRJ and NU groups significantly differed on the number of alcoholic drinks consumed per week, including drinks per week in metabolite analyses as a covariate did not alter the lThal mI/Cr ratio findings. Finally, studies with larger sample sizes may improve the ability to detect additional metabolite alterations in brain regions that are known to be affected by long-term MRJ use.

Total Cr was used to determine metabolite ratios in the current study, which has its own inherent limitations. There is some controversy over the optimal internal reference sample for quantitation of spectral signal intensities. Though Cr levels, a common internal reference, generally reflect little inter-individual variation [33], it is possible that absolute concentrations of Cr may differ under pathological conditions or possibly due to overlap between the Cr signal and other compounds [48]. Thus, Cr was used as an internal reference only after analyses indicated that ratios of Cr/total signal did not differ between groups in any region except in the rTC. Metabolite data from the rTC were generally of insufficient quality, due to signal loss, which has been reported as a potential outcome in imaging medial temporal lobe regions that are particularly subject to differences in magnetic susceptibility [49]. It is possible in the present study that total signal intensity in the rTC was inconsistent across participants, which may reflect individual variability in temporal lobe signal distortion and loss that is in line with previous reports [49]. Subsequent analyses revealed that there were no significant differences in any ratios of metabolites divided by the Cr integral in the rTC. No group differences were observed in the number of voxels with high spectral quality or in the overall tissue percentages across all voxels, suggesting that it is improbable that the lThal-specific mI/Cr reduction in MRJ users was moderated by these measures. Lastly, the self-reported measures of impulsivity and mood state, in addition to drug use, collected in the present study have their own limitations. Self-report can include imprecise or under-reporting of drug use as well as individual differences in response style or interpretation of behavioral or mood questions that are asked in the assessment [50]. Nonetheless, urinary THC/Cr levels provided objective corroboration of self-reported MRJ use in the present study.

In conclusion, this exploratory investigation takes advantage of the ability of MRSI at high field to examine regional specificity of MRJ effects on brain chemistry, and as a result, revealed that young MRJ-dependent men exhibited lower mI/Cr levels in the lThal. Furthermore, these lower lThal mI/Cr levels were a significant neurobiological correlate of greater cognitive impulsivity in MRJ users, reflecting associations that were distinct from those measured in non-MRJ-using age-matched study participants. The present regional findings support earlier work in the same study cohort, which demonstrated significantly greater global mI/Cr levels and less distribution of this glial metabolite in WM in young MRJ-dependent men relative to non-MRJ-using participants. Overall, findings suggest that the lThal may be uniquely vulnerable to long-term MRJ use, and that alterations in lThal mI/Cr levels may influence thalamo-cortical signaling, and in turn, left-lateralized cognitive inhibition circuitry. Furthermore, the present finding of localized metabolite alterations to the lThal indicates that this region may be distinctly impacted by long-term MRJ use and is associated with cognitive impulsivity. Thus, future functional and neurobiological imaging studies, as well as neuropsychological assessments conducted in healthy and MRJ-using populations will be imperative for elucidating the physiological significance of the current exploratory findings.

## Figures and Tables

**Table 1:** Regional proton metabolite ratios.

	Cr/TotSig		Cho/Cr		Glu/Cr		Gln/Cr		mI/Cr		NAA/Cr		NAA+NAAG/Cr	
	NU	MRJ	NU	MRJ	NU	MRJ	NU	MRJ	NU	MRJ	NU	MRJ	NU	MRJ
**lThal**	0.09 ± 0.01	0.10 ± 0.01	4.06 ± 0.56	3.79 ± 0.54	1.09 ± 0.34	0.97 ± 0.09	0.26 ± 0.13	0.20 ± 0.07	1.36 ± 0.31	1.0 ± 0.14 *	1.41 ± 0.23	1.41 ± 0.15	1.85 ± 0.26	1.84 ± 0.24
**rTHal**	0.09 ± 0.01	0.10 ± 0.01	4.06 ± 0.51	3.78 ± 0.43	1.11 ± 0.27	1.04 ± 0.33	0.33 ± 0.19	0.23 ± 0.11	1.25 ± 0.25	1.07 ± 0.18	1.37 ± 0.29	1.2 ± 0.19	1.73 ± 0.36	1.61 ± 0.23
**Thal**	0.35 ± 0.05	0.36 ± 0.04	4.06 ± 0.51	3.8 ± 0.47	1.10 ± 0.28	1.05 ± 0.31	0.32 ± 0.18	0.21 ± 0.08	1.30 ± 0.26	1.06 ± 0.18	1.39 ± 0.24	1.29 ± 0.16	1.79 ± 0.29	1.71 ± 0.22
**lTC**	0.10 ± 0.11	0.09 ± 0.20	3.48 ± 0.22	3.43 ± 0.39	1.17 ± 0.33	1.13 ± 0.15	0.36 ± 0.29	0.44 ± 0.26	1.09 ± 0.22	1.11 ± 0.27	1.52 ± 0.28	1.53 ± 0.37	1.88 ± 0.38	1.92 ± 0.46
**rTC**	0.10 ± 0.03	0.10 ± 0.02 *	3.74 ± 0.48	3.63 ± 0.53	1.11 ± 0.71	1.13 ± 0.50	0.58 ± 0.42	0.50 ± 0.25	1.16 ± 0.15	1.01 ± 0.26	0.88 ± 0.30	1.23 ± 0.37	1.08 ± 0.38	1.51 ± 0.39
**TC**	0.10 ± 0.01	0.09 ± 0.02	3.59 ± 0.24	3.55 ± 0.37	1.15 ± 0.34	1.16 ± 0.31	0.46 ± 0.32	0.50 ± 0.22	1.12 ± 0.18	1.12 ± 0.28	1.32 ± 0.37	1.43 ± 0.32	1.63 ± 0.51	1.79 ± 0.41
**POC**	0.11 ± 0.01	0.11 ± 0.01	2.82 ± 0.31	2.87 ± 0.33	1.20 ± 0.28	1.13 ± 0.25	0.27 ± 0.16	0.31 ± 0.19	1.12 ± 0.18	0.99 ± 0.18	1.42 ± 0.25	1.49 ± 0.46	1.65 ± 0.24	1.77 ± 0.47

Data represent Cr/total signal ± SD and average metabolite ratios (/totalCr) ± SD.

* Indicates statistical significance, p=0.05

**Abbreviations:** Cr-creatine; Cho-choline; Mi-myo-Inositol; NAA-*N*-acetyl-aspartate; NAA+NAAG-*N*-acetyl-aspartate + *N*-acetyl-aspartyl-glutamate; Glu-glutamate; Gln-glutamine

**Table 2:** Voxels analyzed and tissue segmentation between groups.

		Voxels		% Gray Matter		% White Matter	
	NU	MRJ	NU	MRJ	NU	MRJ
**lThal**	3.5 ± 1.3	2.6 ± 1.9	39.3 ± 4.2	41.1 ± 5.6	60.7 ± 4.2	58.9 ± 5.6
**rThal**	3.2 ± 1.3	3.2 ± 1.7	38.8 ± 4.4	39.8 ± 4.8	61.2 ± 4.4	60.2 ± 4.8
**Thal**	6.7 ± 2.5	5.8 ± 3.4	39.0 ± 4.3	40.2 ± 5.1	61.0 ± 4.3	59.8 ± 5.1
**lTC**	3.9 ± 2.3	4.0 ± 2.6	45.8 ± 4.2	48.7 ± 3.4	54.2 ± 4.2	51.3 ± 3.4
**rTC**	4.3 ± 2.4	2.8 ± 2.7	49.1 ± 4.5	52.9 ± 3.1	50.9 ± 4.5	47.1 ± 3.1
**TC**	6.5 ± 3.8	6.6 ± 4.2	47.3 ± 4.1	50.0 ± 2.9	52.7 ± 4.1	50.0 ± 2.9
**POC**	6.5 ± 1.8	6.0 ± 3.1	43.9 ± 2.5	43.3 ± 3.5	56.7 ± 3.5	56.1 ± 2.5

Data represent group averages (± SD) of high spectral quality voxels and relative percentages of gray and white matter in each analyzed region

## Abbreviations

ACC: Anterior Cingulate Cortex
BDI: Beck Depression Inventory
BIS: Barratt Impulsiveness Scale
Cho: Choline
Cr: Creatine
CSF: Cerebrospinal Fluid
DLPFC: Dorsolateral Prefrontal Cortex
GABA: Gamma Amino-Butyric Acid
Glu: Glutamate
Gln: Glutamine
GM: Gray matter
mI: myo-Inositol
MRI: Magnetic Resonance Imaging
MRJ: Marijuana
MRS: Magnetic Resonance Spectroscopy
MRSI: Magnetic Resonance Spectroscopic Imaging
NAA: Nacetyl-aspartate
NAAG: N-acetyl-aspartyl-glutamate
NU: Non-using
PANAS: Positive Affect Negative Affect Schedule
PCr: Phosphocreatine
POC: Parieto-occipital Cortex
POMS: Profile of Mood States
SDGM: Subcortical Deep Gray Matter
T: Tesla
TC: Temporal Cortex
TE: Echo Time
Thal: Thalamus
THC: Δ9-tetrahydrocannabinol
TR: Repetition Time
WM: White Matter
